# Recovery of Hindlimb Motor Function Is Accompanied by Lumbar Neural Remodeling After Complete Spinal Cord Transection in Neonatal Mice

**DOI:** 10.3390/biology15141202

**Published:** 2026-07-21

**Authors:** Wanxing Peng, Ran Li, Lulu Zhang, Wenhui Long, Yingying Yang, Meizhi Wang, Li Song, Jing Li

**Affiliations:** 1 Department of Human Anatomy, The College of Basic Medical Sciences, Jinan University, Guangzhou 510632, China; wxpeng@stu2023.jnu.edu.cn (W.P.); 211dk@stu2025.jnu.edu.cn (L.Z.); yyang@stu2024.jnu.edu.cn (Y.Y.); 2 Guangdong-Hongkong-Macau Institute of CNS Regeneration, Jinan University, Guangzhou 510632, China; liran@stu.jnu.edu.cn (R.L.); lwh2000@stu2025.jnu.edu.cn (W.L.); wangmeizhi0212@jnu.edu.cn (M.W.); 3 Experimental Teaching Center of Basic Medicine, School of Basic Medicine and Public Health, Jinan University, Guangzhou 510632, China

**Keywords:** spinal cord injury, developmental plasticity, synaptic remodeling, locomotor recovery, neonatal mouse

## Abstract

Spinal cord injury often causes persistent neurological dysfunction, whereas the developing central nervous system retains greater plasticity. This study investigated whether spontaneous hindlimb motor recovery occurs after complete spinal cord transection in neonatal mice and whether such recovery is accompanied by structural remodeling of the lumbar spinal cord distal to the lesion. C57BL/6J mice underwent complete T9 spinal cord transection at postnatal day 7, and recovery was assessed by Basso Mouse Scale scoring, CatWalk gait analysis, foot-fault testing, immunofluorescence, and electromyography. Compared with adult mice subjected to the same injury, neonatal mice showed progressive partial improvement in hindlimb motor function, including locomotor performance, gait coordination, and foot placement accuracy. Meanwhile, this recovery was accompanied by preservation of lumbar motor neurons and structural changes in the local neural network. Syn^+^ coverage around lumbar motor neurons was relatively preserved in neonatal injured mice compared with adult injured mice; excitatory synaptic input remained increased, and inhibitory input showed a dynamic pattern of early increase followed by later reduction. In addition, myelin-related alterations were less severe, and motor neuron innervation of peripheral muscles (EMG) were relatively preserved in neonatal mice. The results indicate that the restoration of hindlimb motor function after complete spinal cord transection in neonatal mice is associated with structural and functional remodeling of lumbar spinal motor circuits. This includes enhanced synaptic signals around motor neurons and reorganization of the neuromuscular junction.

## Simple Summary

Functional recovery after spinal cord injury (SCI) remains extremely difficult. However, the nervous system of newborn animals may retain a greater natural ability to adapt and reorganize after damage. In this study, we examined whether mice injured shortly after birth could regain some hindlimb movement after complete spinal cord transection and observed the changes in the structure of the lumbar spinal cord that control hindlimb movements. We found that, unlike adult mice, mice injured as newborns gradually recovered part of their hindlimb movements, including BMS, weight support, coordination, and more accurate foot placement. This improvement was accompanied by plasticity of synapses, myelin, and neuromuscular features in the lumbar spinal cord even though no obvious reconnection across the damaged site was detected. These findings suggest that the recovery of hindlimb motor function following complete spinal cord transection in neonatal mice is associated with enhanced synapse formation in lumbar motor neurons, excitatory and inhibitory reorganization in the lumbar circuit, and remodeling of the neuromuscular junction.

## 1. Introduction

Spinal cord injury (SCI) is a devastating neurological disorder that results in severe impairment of motor, sensory, and autonomic functions, imposing a substantial burden on patients and society [1,2,3]. Epidemiological studies indicate that the annual incidence of SCI ranges from approximately 930,000 people worldwide [4]. Despite extensive efforts, effective therapies capable of producing substantial functional recovery after complete SCI, particularly in adults, remain lacking [5]. This therapeutic limitation underscores the importance of understanding endogenous repair mechanisms that may be retained under specific biological conditions [6,7].

Although the regenerative potential of the adult mammalian central nervous system is highly restricted, both clinical observations and experimental studies suggest that the immature nervous system possesses greater plasticity and compensatory capacity [8,9,10]. Previous work in neonatal rodent models has shown that some degree of spontaneous locomotor recovery can occur after SCI [11]. This phenomenon is frequently attributed, with varying degrees of probability, to developmental axon growth, compensatory sprouting, or network refinement associated with pruning [12,13]. However, under a rigorously validated complete transection paradigm, in which long-distance axonal regeneration from supraspinal centers is excluded, it remains unclear whether the local spinal network distal to the lesion can spontaneously reorganize and generate functional motor output on its own. Whereas many studies have focused on axonal regeneration, neuroprotection, or rehabilitation-induced plasticity after incomplete SCI [14,15], comparatively little attention has been paid to the remodeling potential of the distal lumbar spinal cord after complete transection during early development. In particular, the relationships among neuronal preservation, synaptic remodeling, microenvironmental integrity, and refined behavioral recovery remain insufficiently defined.

To address this question, we established and rigorously validated complete T9 spinal cord transection models in both neonatal and adult mice. By physically separating the two cord stumps and preventing tissue reconnection, we minimized potential confounding by residual or regenerated long-tract axons. Using a multidimensional analytical framework that combined behavioral assessment, histomorphometry, synaptic quantification, and electrophysiological recording, we examined whether the developmental stage influences the endogenous remodeling capacity of distal lumbar spinal circuits and whether the spontaneous hindlimb motor recovery is accompanied by such remodeling. We hypothesized that neonatal mice would exhibit structural plasticity in the lumbar spinal cord despite the absence of supraspinal reconnection.

## 2. Materials and Methods

### 2.1. Animals

This study investigated functional recovery after complete spinal cord transection in postnatal day 7 (P7) neonatal mice and adult mice. Healthy female C57BL/6J mice were used in this study. Adult mice were 8- to 9-week-old females weighing 18–20 g. Neonatal mice underwent surgery at P7. In this study, a total of 24 adult female mice and 62 neonatal mice were used. The surgical success rate was 53% in neonatal mice and 75% in adult mice. Sex was controlled prospectively in adult mice and at postinjury 4 weeks in neonatal mice. To reduce sex-related variability and maintain consistency with the adult injury cohort, only female mice were predefined for endpoint behavioral, histological, and electrophysiological analyses. Four weeks after surgery in neonatal mice (18 neonatal female mice and 15 neonatal male mice), 15 male neonatal mice were therefore not excluded based on postoperative recovery or experimental outcomes, but were not included in the endpoint analyses according to this predetermined sex-control design. Animals were randomly assigned to the sham (n = 6, aged 9 weeks), adult injury (n = 12, adult-8w; six animals were used for structural analysis of the lumbar spinal cord, and the remaining six were used for histological staining in the complete transection model.), neonatal at 8 weeks after injury (n = 12, P7-8w; six animals were used for structural analysis of the lumbar spinal cord, and the remaining six were used for histological staining in the complete transection model.), and neonatal at 16 weeks after injury (n = 6, P7-16w) as appropriate. All animals were housed under specific pathogen-free conditions at 22–24 °C with 50–60% relative humidity under a 12-h light/dark cycle and were provided food and water ad libitum. All procedures were approved by the relevant institutional animal ethics committee and were performed in accordance with institutional guidelines for animal care and use. For all experiments, the experimental unit was a single animal.

To minimize potential confounding factors, animals from different groups were maintained under identical housing conditions and handled according to the same standardized procedures. Behavioral assessments and tissue collection were performed at comparable time points and under the same experimental conditions. Where possible, animals from different groups were assessed in an interleaved order to reduce potential order effects. Cage location was not formally randomized, but all animals were housed in the same facility under identical environmental conditions.

### 2.2. Experimental Design

To systematically evaluate locomotor recovery after complete spinal cord transection and establish a rational comparison framework, this study included neonatal and adult mouse cohorts with predefined observation time points. In the neonatal cohort, mice underwent complete T9 spinal cord transection at P7 and were evaluated at 8 weeks (P7-8W) and 16 weeks (P7-16W) after surgery to assess early and long-term recovery, respectively; 16 weeks was selected as the final endpoint because preliminary observations showed that both BMS scores and CatWalk gait parameters had largely stabilized after this time. In the adult cohort, 8- to 9-week-old female mice underwent complete T9 transection and were evaluated 8 weeks later (Adult-8W), allowing comparison with the neonatal cohort at a similar age stage. Sham-operated mice underwent the same anesthesia and surgical exposure without cord transection, and sham mice of different ages were initially examined to assess age-related effects on gait parameters; because both the P7-16W and Adult-8W groups were approximately 17 weeks old at the endpoint, the 17-week sham group was used as the final age-matched control. All surgeries were performed under sterile conditions, animals received routine postoperative care, behavioral assessments were conducted at the predefined endpoints, and mice were acclimatized before CatWalk testing. This design enabled comparison of recovery dynamics within the neonatal cohort, differences in spontaneous recovery between neonatal and adult injury, and functional performance relative to age-matched normal controls.

A study protocol including the research question, key design features, and analysis plan was prepared before the study, but it was not registered in a public repository.

### 2.3. Complete Spinal Cord Transection Model

For adult mice, anesthesia was induced by intraperitoneal injection of 1.25% tribromoethanol (0.2 mL/10 g). After laminectomy, the T9 spinal cord segment was completely excised under a dissecting microscope until the vertebral floor was clearly visible, thereby ensuring full transection. The lesion cavity was filled with an absorbable gelatin sponge to prevent reconnection between the rostral and caudal stumps, and the wound was closed in layers. Postoperatively, mice received antibiotic treatment and manual bladder expression. Tissue was collected 8 weeks after injury. The postoperative survival rate of adult mice was 75%. Sham-operated mice underwent laminectomy alone.

For neonatal mice, P7 pups were anesthetized by hypothermia on ice. The T9 spinal cord segment was exposed and completely removed in the same manner, followed by gelatin sponge implantation and layered closure. Lidocaine gel was applied locally for postoperative analgesia. The postoperative survival rate of neonatal mice operated on at postnatal day 7 (P7) was 53%. Pups were rewarmed until recovery, closely monitored during the first postoperative week, and underwent assisted bladder emptying when necessary. Neonatal SCI mice were analyzed at 8 or 16 weeks after surgery, designated P7-8W and P7-16W, respectively.

Sham-operated mice underwent the same surgical exposure and T9 laminectomy procedure; however, only the vertebral lamina was removed. The spinal cord was left intact and was not subjected to transection, segment removal, compression, or gelatin sponge implantation. Mice in the sham group underwent laminectomy at 9 weeks of age, and data were collected 8 weeks postoperatively.

The protocol for complete spinal cord transection in both adult and neonatal mice, together with the inclusion and exclusion criteria, was established in accordance with previously studies [16,17]. All inclusion and exclusion criteria were predefined prior to study initiation. Animals were considered successfully modelled and included in the study only if they exhibited complete paralysis of both hindlimbs on the first day after surgery. Animals were excluded if spinal cord transection was incomplete on histological verification, if they failed to meet the predefined postoperative motor criterion, if they died before the intended endpoint, or if severe postoperative complications interfered with reliable outcome assessment.

### 2.4. Behavioral Assessment

Hindlimb locomotor function was assessed using the Basso Mouse Scale (BMS), CatWalk automated gait analysis, and the foot-fault test at predefined postoperative time points. All behavioral evaluations were performed during the light cycle by investigators blinded to group allocation.

Basso Mouse Scale (BMS):

Open-field locomotor function was evaluated using the Basso Mouse Scale (BMS), a 9-point locomotor rating system specifically developed for mice after SCI. Mice were individually placed in a circular open field (90 cm diameter) and allowed to move freely for 4 min. Locomotor performance was recorded using a digital video camera. Two independent observers, blinded to the experimental groups, scored each animal according to the criteria established by Basso et al. [18], including ankle movement, plantar stepping, forelimb–hindlimb coordination, trunk stability, and tail position. The average score from the two observers was used for statistical analysis.

CatWalk Automated Gait Analysis:

Quantitative gait analysis was performed using the CatWalk XT system (Noldus Information Technology, Wageningen, The Netherlands). Mice were trained to traverse the illuminated glass walkway before data collection. During testing, animals were allowed to voluntarily cross the walkway without interruption. Runs with pauses, changes in direction, or grooming behavior were excluded from analysis. For each animal, at least three compliant runs were recorded and averaged.

The CatWalk software XT 10.7 automatically quantified multiple gait parameters, including maximal contact area, stride length, regularity index, print area, swing duration, stance duration, duty cycle, and hind-paw coordination parameters. To minimize the influence of body size differences among age groups, maximal contact area and contact max intensity were normalized to body weight when appropriate. All analyses were conducted using CatWalk software XT 10.7 according to the manufacturer’s instructions [19].

Foot-Fault Test:

Fine motor coordination and paw placement accuracy were assessed using the foot-fault test [20]. Mice were placed on an elevated metal grid (mesh size: 1 cm × 1 cm) and allowed to freely explore for 3 min. A foot fault was defined as an instance in which a hind paw slipped completely through the grid during locomotion. The total number of hindlimb steps and the number of foot faults were recorded from video footage by blinded observers.

The foot-fault rate was calculated as:

Foot-fault rate (%) = (Number of foot faults/Total number of hindlimb steps) × 100 For each animal, three trials were performed with at least 5 min of rest between trials, and the mean value was used for subsequent statistical analysis.

### 2.5. Immunofluorescence and Quantitative Image Analysis

Animal perfusion and sample processing:

At the designated endpoints, mice were deeply anesthetized and transcardially perfused with phosphate-buffered saline (PBS), followed by 4% paraformaldehyde (PFA). Spinal cord segments located 2 mm rostral and 2 mm caudal to the lesion, encompassing the lesion site itself in some mice (n = 6) were dissected. Gastrocnemius muscles were dissected from both hindlimbs and collected from tendon to tendon with minimal mechanical manipulation (n = 6). Lumbar spinal cord segments (L1–L5) distal to the injury site in other animals (n = 6) were carefully dissected. These tissues were fixed in 4% paraformaldehyde overnight at 4 °C, followed by cryoprotection in 30% sucrose solution and embedding in OCT. Spinal lesion segments were sectioned longitudinally at a thickness of 20 μm using a cryostat. Horizontal cryosections (20 μm) were prepared for NMJ analysis. Lumbar spinal cord segments were sectioned coronally at a thickness of 15 μm using a cryostat.

The staining and analysis of gastrocnemius muscles:

NMJs were labeled using fluorescent α-bungarotoxin (α-BT), which selectively binds postsynaptic acetylcholine receptors (AChRs). Sections were permeabilized with 0.3% Triton X-100 and incubated with α-BT conjugated fluorophore at 4 °C overnight. After washing, slides were mounted using antifade medium. Confocal images were acquired under identical settings across all groups, including constant laser power, gain, offset, and detector sensitivity. Images were collected from randomly selected fields within the mid-belly region of the gastrocnemius muscle. NMJ morphology was quantified using ImageJ (National Institutes of Health, Bethesda, MD, USA; version 1.53t). AChR cluster number and area were measured using a fixed threshold applied uniformly across all samples. Fragmented AChR clusters were defined as discontinuous, split, or perforated postsynaptic plaques. The fragmentation rate was calculated as the percentage of fragmented AChRs relative to total NMJs analyzed per animal. All analyses were performed under blinded conditions.

The staining and analysis of spinal lesions and lumbar spinal cord segments:

Longitudinally consecutive sections were stained with NF200 and GFAP. After washing in PBS, sections were incubated with species-specific fluorescent secondary antibodies for 1 h at room temperature and counterstained with DAPI. Negative controls omitting the primary antibodies were included to verify staining specificity. Fluorescence images were acquired using a laser-scanning confocal microscope under identical acquisition settings for all groups within each staining batch. Laser intensity, detector gain, pinhole size, and exposure parameters were kept constant throughout image acquisition to enable quantitative comparisons.

To ensure systematic sampling throughout the lumbar enlargement, serial sections were collected into five parallel sets. Coronally consecutive sections were assigned sequentially to Set 1–Set 5, such that each set represented the entire rostro-caudal extent of the lumbar spinal cord while maintaining an interval of approximately 80 μm between adjacent sections within the same set. Immunofluorescence staining was performed on all five sets of sections from each animal. After blocking with 5% normal donkey serum containing 0.3% Triton X-100 for 1 h at room temperature, sections were incubated overnight at 4 °C with primary antibodies against choline acetyltransferase (ChAT, motor neurons), βIII-tubulin (Tuj1, axons and dendrites), synaptophysin (Syn, total synapses), vesicular glutamate transporter 1 (VGLUT1, excitatory presynaptic terminals), vesicular GABA transporter (VGAT, inhibitory presynaptic terminals), and myelin basic protein (MBP, myelin).

For ChAT staining, the number of motor neurons and motor neuron density were quantified within the ventral horn. For Tuj1 staining, the percentage area occupied by Tuj1-positive fibers was measured as an index of axonal and dendritic density. Syn^+^, VGLUT1^+^, and VGAT^+^ puncta directly covered by ChAT-positive motor neuron soma were quantified using high-magnification confocal images. Synaptic density was calculated as the number of immunoreactive puncta normalized to the perimeter of individual motor neuron somata (puncta/μm). For MBP staining, mean fluorescence intensity and MBP-positive area fraction in the lumbar ventral horn around ChAT-positive motor neurons were quantified to assess myelin content.

Tuj1 immunofluorescence images of lumbar spinal cords (L1–L5) were acquired using a confocal microscope under identical settings across all groups. For each animal, ventral horn regions were randomly selected for analysis. Z-stack images were acquired and processed as maximum intensity projections. When single-plane analysis was used, the optical section with the best signal-to-noise ratio was selected consistently across samples. Images were processed in ImageJ (National Institutes of Health, Bethesda, MD, USA; version 1.53t). Background subtraction was performed using a fixed rolling-ball radius. Tuj1-positive structures were segmented using a fixed intensity threshold determined from sham controls and applied to all groups. Neurite analysis was performed after skeletonization using the ImageJ “Skeletonize” function followed by the “Analyze Skeleton” plugin. Total neurite length, branch number, and junctions were quantified. All parameters were kept constant, and no manual editing was performed. Images with tissue damage, saturation, strong auto-fluorescence, or out-of-focus artifacts were excluded before analysis. All analyses were performed in a blinded manner. All measurements were normalized to field area, and identical processing pipelines were used for all groups.

For quantitative analysis, regions of interest (ROIs) were defined within the lumbar ventral horn based on anatomical landmarks. All image analyses were performed using ImageJ software (NIH, USA) by investigators blinded to experimental group allocation. For each animal, images were obtained from all five serial section sets. Quantitative values from multiple matched sections within each set were first averaged to generate a single value for that set. The mean value of the five sets was then calculated to obtain one representative value per animal. Six animals were included in each experimental group, and individual animals rather than individual sections were considered the statistical unit for all analyses.

### 2.6. Electromyography

Sciatic nerve-evoked electromyography (EMG) recordings were performed at the designated experimental endpoints. Mice were anesthetized with isoflurane (induction: 3%; maintenance: 1.5–2.0%) and placed on a thermostatically controlled heating pad to maintain body temperature at approximately 37 °C throughout the recording procedure. The left sciatic nerve was surgically exposed at the mid-thigh level through a small skin incision and carefully separated from the surrounding connective tissue. Electrical stimulation was delivered to the sciatic nerve using a bipolar hook electrode connected to a constant-current stimulator. Square-wave pulses (0.1 ms duration) were applied at supramaximal intensity (typically 2–5 mA) to ensure reliable activation of motor axons. Stimuli were delivered at a frequency of 1 Hz. Compound muscle action potentials (CMAPs) were recorded using sterile monopolar needle electrodes inserted into the ipsilateral gastrocnemius muscle. A reference electrode was placed subcutaneously near the Achilles tendon, and a ground electrode was positioned in the tail. EMG signals were amplified, band-pass filtered (10–3000 Hz), digitized, and recorded using an electrophysiological acquisition system. For each animal, at least ten consecutive responses were recorded, and the average waveform was used for quantitative analysis. The onset latency was defined as the interval between stimulus delivery and the initial deflection of the CMAP from baseline. Peak-to-peak amplitude was measured as the voltage difference between the maximal positive and negative peaks of the evoked response. Latency was used as an indicator of neuromuscular conduction efficiency, whereas CMAP amplitude reflected the functional integrity of motor axons, neuromuscular transmission, and muscle activation. All recordings and analyses were performed under identical experimental conditions by investigators blinded to group allocation.

### 2.7. Statistical Analysis

Data were presented as the mean ± SD. Statistical analyses were performed using GraphPad Prism 10.0. For histological and immunofluorescence quantification, multiple sections and/or imaging fields from each animal were averaged first, and each animal contributed one value to the statistical analysis. Exact n values are provided in the corresponding figure legends. Normality was assessed before group comparisons. For comparisons among more than two independent groups, one-way ANOVA followed by Tukey’s multiple-comparisons test was used for normally distributed data, whereas Kruskal–Wallis tests followed by Dunn’s multiple-comparisons correction were used for non-normally distributed data. Longitudinal BMS scores were analyzed using a two-way repeated-measures ANOVA with group and time as factors, followed by Šídák’s multiple-comparisons correction. When missing values were present, mixed-effects models were used. Longitudinal CatWalk parameters and foot-fault miss rates were analyzed using mixed-effects models with post-injury time as a repeated factor, followed by Tukey’s multiple-comparisons test. Pearson correlation analysis was used to assess the association between left and right hindlimb duty cycle. Multiple testing was corrected within related endpoint families, including behavioral outcomes, CatWalk gait parameters, EMG parameters, neuromuscular junction morphology, ChAT/Tuj1 morphology, synaptic markers, myelin-related endpoints, and VGLUT1/VGAT puncta-density endpoints. The Holm–Šídák correction was used for multiple testing across related endpoints, unless otherwise specified. *p* < 0.05 was considered statistically significant. ns, not significant; * *p* < 0.05; ** *p* < 0.01; *** *p* < 0.001; **** *p* < 0.0001.

### 2.8. Randomization and Blinding

Animals were assigned to experimental groups before surgery. Due to the clear differences in body size, developmental stage, and surgical handling between neonatal and adult mice, complete blinding during model preparation was not feasible, and the surgeon was aware of the experimental allocation, and the cage location was not formally randomized. However, behavioral assessment, gait analysis, tissue processing, immunohistochemical/immunofluorescence staining, image acquisition, quantitative analysis, and statistical analysis were performed by investigators blinded to group allocation whenever feasible.

## 3. Results

### 3.1. Histological and Structural Analysis of the Lesion Site After Complete T9 Spinal Cord Transection

**Figure 1:**
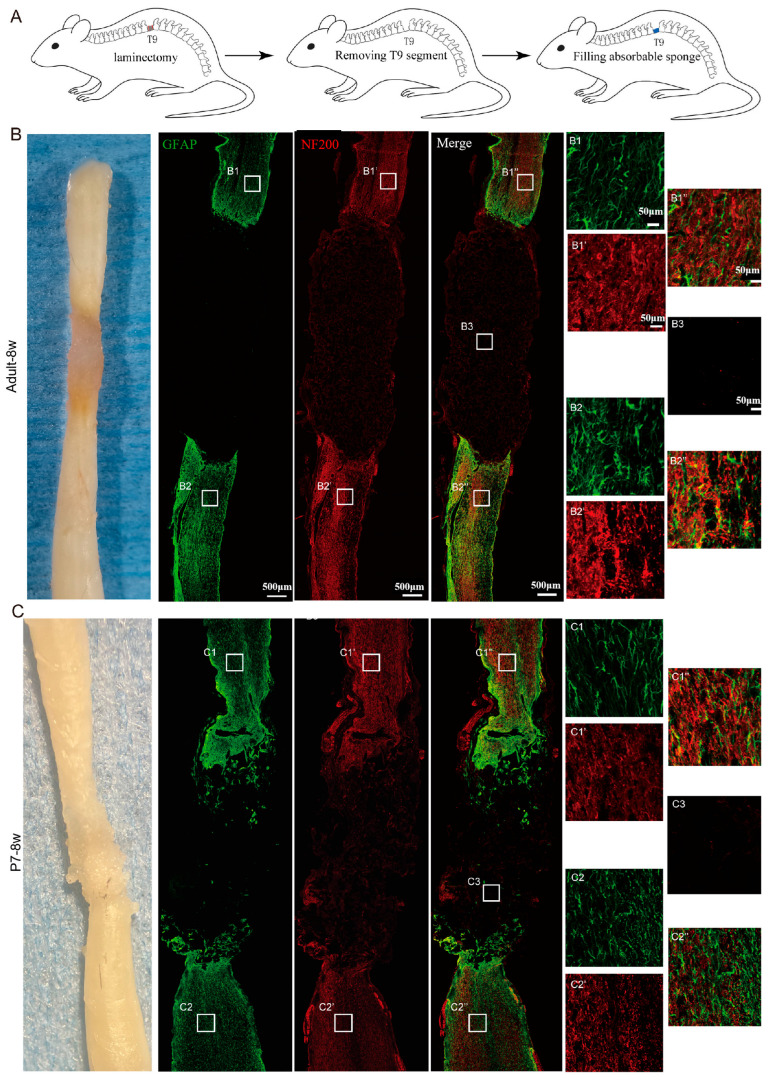
Histological analysis of the lesion site after complete T9 spinal cord transection in adult and neonatal mice. (**A**) Schematic illustration of the complete T9 spinal cord transection procedure. After T9 laminectomy, the T9 spinal cord segment was removed to generate a complete transection gap, followed by filling of the lesion cavity with an absorbable gelatin sponge to prevent spontaneous reconnection between the rostral and caudal stumps. (**B**) Representative and longitudinal immunofluorescence images of the adult spinal cord after complete T9 transection. GFAP staining (Green) labels astrocytes, and NF200 staining (Red) labels neurofilament-positive axons. The rostral and caudal spinal cord stumps are clearly separated by a lesion gap, with no continuous NF200-positive axonal bridge across the transection site. High-magnification images of selected regions show GFAP- and NF200-positive structures in the rostral stump (B1/B1′) and caudal stump (B2/B2′), whereas the central lesion gap (B3) contains no neural signal. (**C**) Representative and longitudinal immunofluorescence images of the spinal cord 8 weeks after complete T9 transection performed at postnatal day 7. Similar to the Adult-8w, the rostral and caudal stumps remain anatomically separated, and no continuous NF200-positive axonal structure is observed across the lesion cavity. High-magnification images show GFAP- and NF200-positive staining in the rostral stump (C1/C1′) and caudal stump (C2/C2′), while the central lesion region (C3) shows no neural continuity. Scale bars: 500 μm in 20× magnification spinal cord images; 50 μm in 40× magnification insets. n = 6.

To evaluate the completeness of the T9 spinal cord transection, we performed longitudinal immunofluorescence analysis across the lesion site. As shown in Figure 1A, after T9 laminectomy, the T9 spinal cord segment was removed, and the lesion gap was filled with an absorbable gelatin sponge. Longitudinal sections across the lesion region were stained for GFAP and NF200. In Adult-8W, GFAP-positive astrocytic signals were mainly distributed at the rostral and caudal spinal cord stumps, while the central lesion gap showed minimal GFAP-positive signal. And NF200-positive axonal structures were abundant within the rostral and caudal stumps but did not form a continuous bridge across the lesion gap (Figure 1B). High-magnification images further confirmed the presence of GFAP- and NF200-positive structures in the rostral and caudal stumps, whereas the central gap region showed very little NF200 signal and no obvious neural continuity. Similarly, in the P7-8W group, the rostral and caudal spinal cord stumps remained clearly separated by a lesion cavity. GFAP-positive signals were mainly detected around the stump borders, consistent with astrocytic responses at the host tissue margins. And NF200 staining was present within the rostral and caudal spinal cord tissue but was discontinuous across the transection site (Figure 1C). High-magnification views of the rostral stump, caudal stump, and central gap region confirmed the absence of a continuous NF200-positive axonal bridge. Together, GFAP/NF200 immunofluorescence data demonstrate that the T9 spinal cord segment was completely removed, and that no obvious spared or reconnected axonal fibers were detected across the lesion gap in either the adult or neonatal transection models.

### 3.2. Neonatal Mice Exhibited Substantial Spontaneous Hindlimb Motor Recovery After Complete SCI

#### 3.2.1. Hindlimb Motor Performance Progressively Improved in Neonatal Mice After SCI

**Figure 2:**
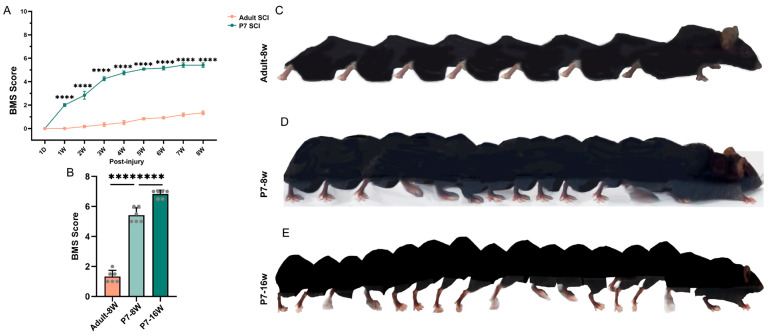
Progressive recovery of hindlimb motor function in P7 mice. (**A**) Time-course analysis of BMS scores from 1 day to 8 weeks after complete T9 spinal cord transection in P7-SCI and Adult-SCI mice. P7-SCI mice showed partial improvement in hindlimb motor function, whereas Adult-SCI mice remained severely impaired during the same observation period. (**B**) Quantification of BMS scores in the Adult-8W, P7-8W, and P7-16W mice. P7-SCI mice showed significantly higher BMS scores than Adult-SCI mice, with further improvement from the P7-8W to P7-16W. (**C**–**E**) Representative sequential locomotor images showing hindlimb movement patterns in the Adult-8W, P7-8W, and P7-16W mice. Data are presented as mean ± SD. For longitudinal BMS scores in panel A, data were analyzed using a two-way repeated-measures ANOVA with group and time as factors, followed by the Šídák’s multiple-comparisons test for between-group comparisons at each time point. For endpoint BMS scores in panel B, comparisons among the Adult-8W, P7-8W, and P7-16W groups were performed using one-way ANOVA followed by Tukey’s multiple-comparisons test. n = 6, **** *p* < 0.0001.

Following complete T9 transection, P7 mice showed spontaneous and time-dependent recovery of hindlimb motor function (Figure 2A). BMS scores increased progressively after injury, and by 3 weeks postoperatively, the mice were able to achieve bilateral hindlimb support and generate stepping behavior. By 8 weeks after injury, BMS scores reached approximately 5 points, indicating spontaneous locomotor improvement. In contrast, adult mice subjected to the same complete transection paradigm remained at persistently low BMS levels (below 1 point) throughout the 8-week observation period. The difference between the neonatal and adult groups was statistically significant across the postoperative time course (Figure 2A). Further comparison among the Adult-8W, P7-8W, and P7-16W groups showed that both the P7-8W and P7-16W groups exhibited significantly better locomotor recovery than the Adult-8W group (Figure 2B–E). Notably, BMS scores were further increased in the P7-16W compared with the P7-8W. Consistent with the quantitative BMS analysis, representative sequential locomotor images showed limited hindlimb movement in the Adult-8W, whereas animals of the P7-8W restored partial hindlimb stepping ability. Together, these results indicate that complete spinal cord transection at P7 is followed by partial spontaneous recovery of hindlimb locomotor function, whereas adult mice subjected to the same injury remain severely impaired.

#### 3.2.2. Baseline Gait Parameters Became Stable with Age in Sham Controls

**Figure 3:**
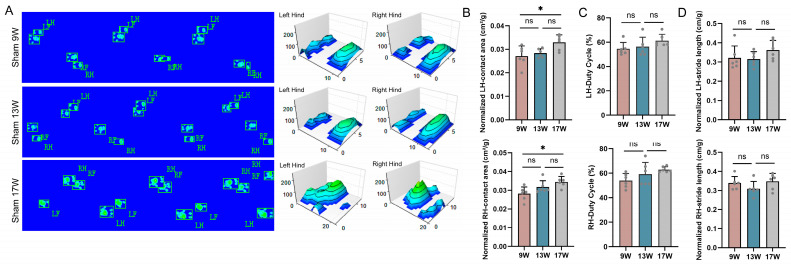
Comparison of gait parameters in the Sham group at 9, 13, and 17 weeks. (**A**) CatWalk gait footprints (**left**) and real-time hindlimb pressure distribution during locomotion (**right**) in Sham mice at 9, 13, and 17 weeks. (**B**–**D**) Quantitative analysis of left and right hindlimb gait parameters, including maximum contact area (**B**), duty cycle (**C**), and stride length (**D**). Data are presented as mean ± SD. Comparisons among Sham 9W, Sham 13W, and Sham 17W groups were performed using one-way ANOVA followed by Tukey’s multiple-comparisons test. For datasets that did not meet the assumptions of normality, Kruskal–Wallis tests followed by Dunn’s multiple-comparisons correction were used. To account for multiple CatWalk gait-parameter comparisons, adjusted *p* values were further controlled within the CatWalk analysis family using the Holm-Šídák correction. n = 6, * *p* < 0.05; ns, no significant difference.

To distinguish injury-related gait changes from normal developmental maturation, gait parameters in the CatWalk Test were compared among sham animals at different ages (Figure 3A). The maximal contact area increased mildly with age, whereas normalized stride length and duty cycle remained largely stable from juvenile to adult stages (Figure 3B–D). These findings indicate that, after normalization, gait rhythm and stance-swing coordination remain relatively stable across the relevant age window and therefore provide an appropriate baseline for interpreting postoperative locomotor changes in SCI mice.

#### 3.2.3. Hindlimb Weight-Bearing Capacity Gradually Recovered After Neonatal SCI

**Figure 4:**
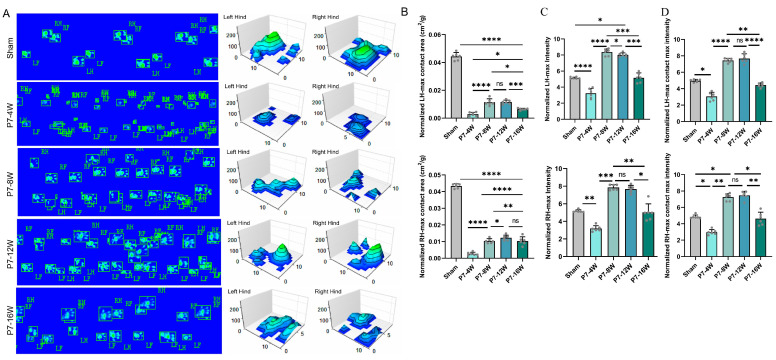
Changes in hindlimb weight-bearing and contact ability after complete spinal cord transection in P7 mice. (**A**) CatWalk gait footprints (**left**) and real-time hindlimb pressure distribution during locomotion (**right**) in the Sham (underwent laminectomy at 9 weeks of age, and data were collected 8 weeks postoperatively) and P7 mice at different postoperative time points. (**B**–**D**) Analysis of hindlimb weight-bearing–related parameters, including the normalized maximum contact area of the left and right hindlimbs (**B**), normalized maximum intensity (**C**), and the normalized maximum contact intensity (**D**). Data are presented as the mean ± SD. For CatWalk gait parameters measured longitudinally after P7 transection, data were analyzed using a mixed-effects model with time as a repeated factor, followed by Tukey’s multiple-comparisons test. Sham mice were included as the reference control group for baseline gait performance. For each CatWalk parameter, the left and right hindlimbs were analyzed separately. To account for multiple testing across related CatWalk parameters, *p* values were further adjusted within the CatWalk analysis family using the Holm-Šídák correction. n = 6, * *p* < 0.05, ** *p* < 0.01, *** *p* < 0.001, **** *p* < 0.0001; ns, not significant.

CatWalk analysis demonstrated a marked postoperative increase in hindlimb load-bearing function in P7 mice (Figure 4A). At 4 weeks after injury, the maximal contact area was dramatically reduced, indicating severely impaired hindlimb support. As recovery progressed, maximal contact area increased substantially at 8 and 12 weeks, with a significant improvement between these time points, reflecting progressive restoration of weight-bearing ability. By 16 weeks, the contact area was higher than 8 and 12 weeks (Figure 4B). Similar trends were observed for maximal intensity (Figure 4C) and weighted contact intensity (Figure 4D), supporting the conclusion that neonatal mice gradually regained partial hindlimb loading after complete SCI.

#### 3.2.4. Gait Coordination Progressively Improved During Recovery

**Figure 5:**
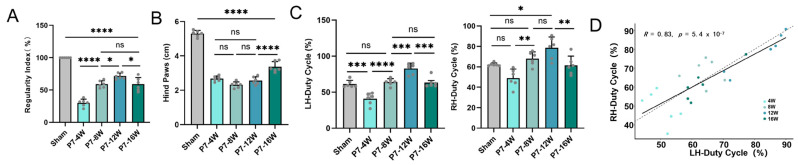
Progressive improvement of hindlimb gait coordination and rhythm in P7 mice after complete spinal cord transection. (**A**) Changes in hindlimb gait regularity index (Regularity Index, RI) over time during recovery. (**B**) Quantitative analysis of hind paw stepping parameters (Hind Paws). (**C**) Changes in duty cycle of the left and right hindlimbs over time during recovery. (**D**) Correlation analysis of duty cycle between the left and right hindlimbs by Pearson correlation analysis, and using the mean duty cycle of each animal at each time point. Data are presented as the mean ± SD. Longitudinal CatWalk gait parameters were analyzed using a mixed-effects model with time as a repeated factor, followed by Tukey’s multiple-comparisons test. Multiple testing across related CatWalk parameters was controlled using the Holm–Šídák correction. Pearson correlation analysis was used for LH-duty cycle and RH-duty cycle association analysis. n = 6, * *p* < 0.05, ** *p* < 0.01, *** *p* < 0.001, **** *p* < 0.0001, ns indicates no significant difference.

To assess locomotor coordination, the regularity index and hind-paw stepping parameters were analyzed in the CatWalk test. Neonatal mice subjected to complete spinal cord transection at P7 showed progressive improvement in gait coordination over time. Quantitative analysis revealed that the regularity index was markedly reduced at 4 weeks post-injury, but significantly increased at 8 and 12 weeks, indicating gradual recovery of interlimb coordination; although this recovery remained incomplete compared with the sham group (Figure 5A). Consistently, hind paw performance was severely impaired at 4 weeks and progressively improved thereafter, with significantly higher scores at 16 weeks post-injury (Figure 5B). Analysis of gait phase relationships further showed that the proportion of left hindlimb–right forelimb (LH-RF) coupling increased over time, whereas right hindlimb–left forelimb (RH-LF) coupling also exhibited partial restoration, particularly at later time points (Figure 5C). Importantly, the regularity index was strongly positively correlated with the LH duty cycle, suggesting that improved temporal coordination of hindlimb movement contributes substantially to locomotor recovery after neonatal complete transection (Figure 5D). Together, these data indicate that neonatal mice gradually re-established coordinated locomotor output after complete SCI.

#### 3.2.5. Forelimb Activity Was Not Sufficient to Explain Hindlimb Recovery

**Figure 6:**
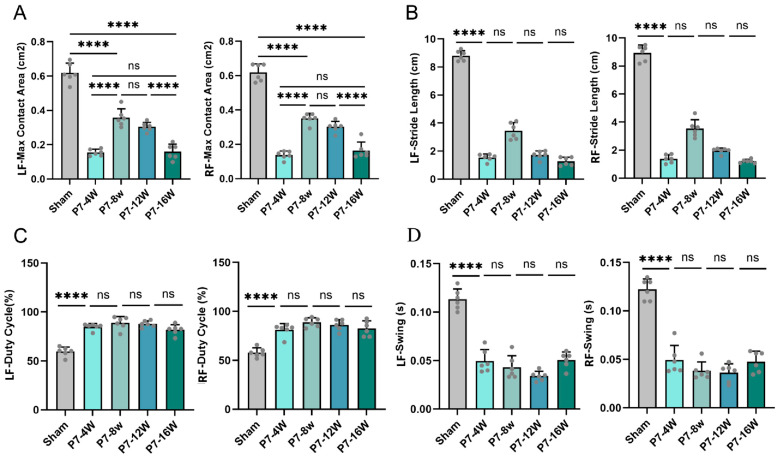
Forelimb gait analysis in P7 mice after complete spinal cord transection. (**A**–**D**) Changes in gait parameters of the left and right forelimbs, including maximum contact area (Max Contact Area) (**A**), stride length (Stride Length) (**B**), duty cycle (Duty Cycle) (**C**), and swing (Swing) (**D**). Data are presented as mean ± SD. Longitudinal CatWalk gait parameters were analyzed using a mixed-effects model with post-injury time as a repeated factor, followed by Tukey’s multiple-comparisons test. Multiple testing across related CatWalk parameters was controlled using the Holm–Šídák correction. n = 6, **** *p* < 0.0001, ns indicates no significant difference.

To determine whether forelimb locomotion contributed to hindlimb recovery, forelimb gait parameters were analyzed in parallel. In sham mice, forelimb gait parameters remained stable and consistent with normal coordinated walking. In P7 mice, forelimb parameters showed some temporal fluctuation but remained significantly lower than those of sham controls, and no clear stage-dependent improvement was observed (Figure 6). Moreover, changes in forelimb activity did not mirror the recovery trajectory of hindlimb gait. These findings suggest that the spontaneous partial hindlimb locomotor improvement observed after complete transection in neonatal mice was not primarily induced by forelimb compensation.

#### 3.2.6. Fine Hindlimb Motor Control Gradually Recovered After Neonatal SCI

**Figure 7:**
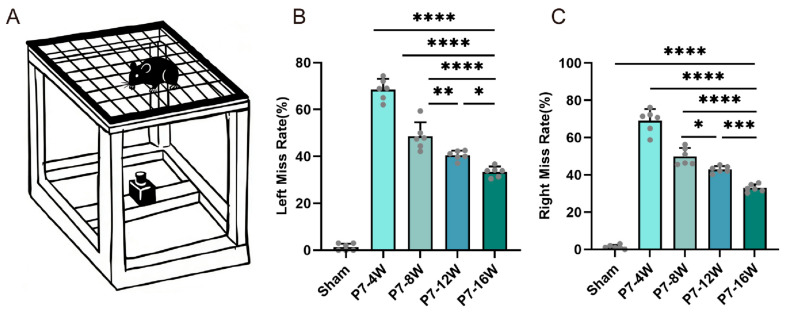
Foot Fault test assessing the recovery of hindlimb fine motor function in P7 mice after complete spinal cord transection. (**A**) Schematic diagram of the Foot Fault test. (**B**) Foot fault rate (%) of the left hindlimb. (**C**) Foot fault rate (%) of the right hindlimb. Data are presented as mean ± SD. Left and right hindlimb miss rates were analyzed using a mixed-effects model with post-injury time as a repeated factor, followed by Tukey’s multiple-comparisons test. When all animals had complete measurements across all time points, repeated-measures one-way ANOVA was used. Multiple testing across left and right miss-rate analyses was controlled using the Holm–Šídák correction. n = 6, * *p* < 0.05, ** *p* < 0.01, *** *p* < 0.001, **** *p* < 0.0001.

The foot-fault test showed that the sham mice maintained very low error rates, indicating precise hindlimb placement during locomotion (Figure 7A). In contrast, the P7 group exhibited the highest foot-fault rate at the early postoperative stage, reflecting marked impairment of fine motor control. As recovery progressed, foot-fault rates gradually declined in both hindlimbs, demonstrating progressive restoration of paw placement accuracy. Error rates of neonatal mice with SCI remained higher than those of the sham mice at all time points (Figure 7B,C), which indicates that local lumbar circuits in neonatal mice partially regained the capacity to support fine motor coordination after complete SCI.

### 3.3. Neonatal Spinal Transection Promotes NMJ Remodeling in the Gastrocnemius Muscle

**Figure 8:**
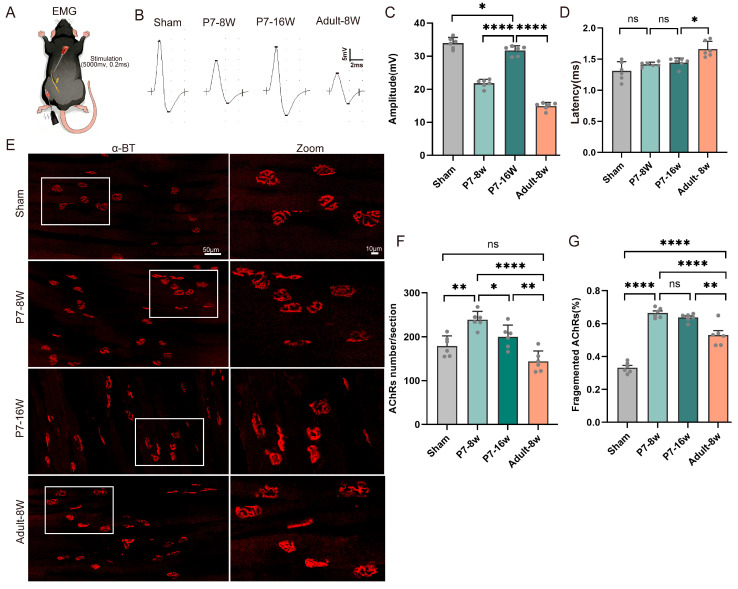
Analysis of neuromuscular junction function and structure in adult and neonatal mice after complete spinal cord transection. (**A**) Schematic diagram of the sciatic nerve–muscle electrophysiological experiment. (**B**) Changes in action potentials during spontaneous recovery after T9 complete spinal cord transection. (**C**,**D**) Changes in action potential amplitude (**C**) and latency (**D**) during spontaneous recovery in four groups. (**E**) α-Bungarotoxin (α-BT) staining of the hindlimb gastrocnemius muscle in four groups. (**F**,**G**) Quantification of α-BT in the hindlimb gastrocnemius muscle, including number (**F**) and fragmentation rate (**G**), in four groups. Data are presented as mean ± SD. Comparisons among groups were performed using one-way ANOVA followed by Tukey’s multiple-comparisons test, and multiple testing across related EMG and NMJ endpoints was controlled using the Holm–Šídák correction. n = 6, * *p* < 0.05, ** *p* < 0.01, **** *p* < 0.0001.

To assess whether locomotor recovery in neonatal mice after complete transection was accompanied by preservation of the neuromuscular junction, we performed electrophysiological and neuromuscular junction analyses. Evoked recordings showed that signal amplitude was significantly reduced in the P7-8W group but was partially restored in the P7-16W group, reaching levels higher than those in the Adult-8W group (Figure 8B,C). In contrast, response latency remained unchanged among the sham, P7-8W, and P7-16W groups, whereas the Adult-8W group showed a significant increase in latency (Figure 8D). Consistent with the electrophysiological findings, α-BT labeling of acetylcholine receptors (AChRs) revealed marked remodeling of neuromuscular junctions after injury (Figure 8E). Quantification showed that the total AChR number was increased in the P7-8W group, and remained higher than that in the P7-16W group (Figure 8F). Moreover, the proportion of fragmented AChRs was significantly elevated in both the P7 and adult mice with SCI compared with the sham group, indicating persistent postsynaptic remodeling after transection (Figure 8G). Together, these data suggest that neonatal mice following SCI preserve a capacity for neuromuscular reorganization than adult mice.

### 3.4. Neonatal Mice Following SCI Preserve Spinal Motor Neurons but Is Associated with Profound Remodeling of Local Neurite Architecture

**Figure 9:**
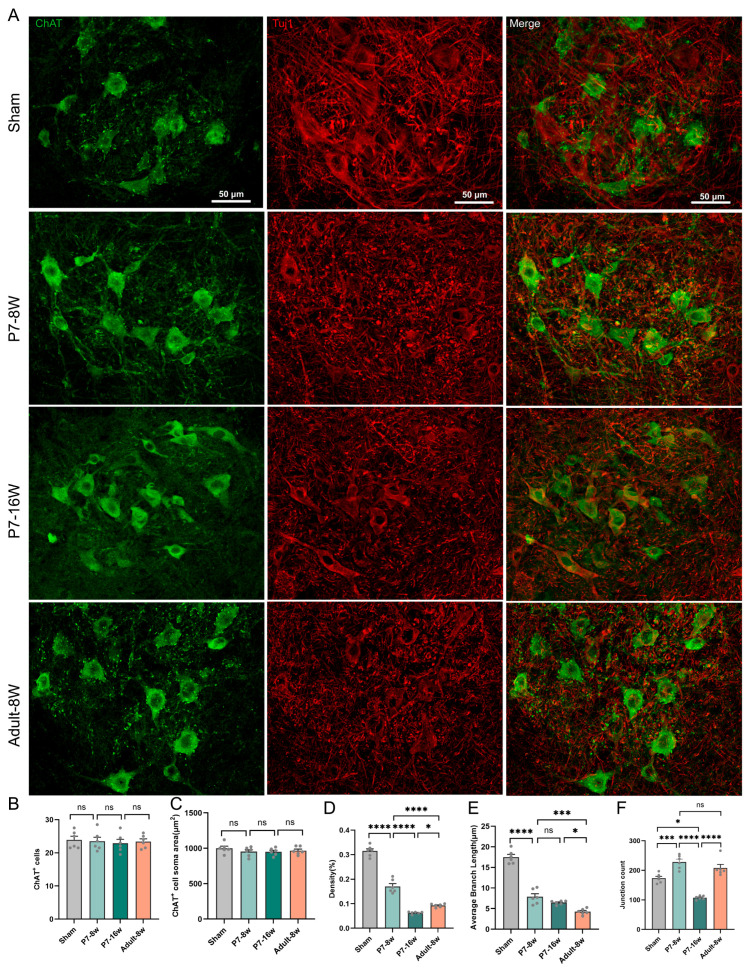
Analysis of lumbar motor neuron and axonal structural remodeling. (**A**) Representative images of ChAT (Green) and Tuj1 (Red) immunofluorescence co-labeling in the lumbar spinal cord of Sham, P7-8W, P7-16W, and Adult-8W groups. (**B**,**C**) Quantitative analysis of ChAT-positive motor neurons in the lumbar spinal cord: number of ChAT-positive neurons (**B**) and soma area of ChAT-positive neurons (**C**). (**D**–**F**) Quantitative analysis of neurite structures within the regions of ChAT-positive motor neurons in the lumbar spinal cord, including Tuj1 signal density (**D**), average branch length (**E**), and number of branch junctions (**F**). Data are presented as mean ± SD. Comparisons among groups were performed using one-way ANOVA followed by Tukey’s multiple-comparisons test, and multiple testing across related ChAT/Tuj1 morphological endpoints was controlled using the Holm–Šídák correction. n = 6, * *p* < 0.05, *** *p* < 0.001, **** *p* < 0.0001, ns indicates no significant difference.

To determine whether spontaneous locomotor recovery in neonatal mice after SCI was associated with changes in lumbar motor neurons, we examined ChAT and Tuj1 labeling in the distal spinal cord. Quantification showed no significant differences in the number of ChAT^+^ motor neurons or in ChAT^+^ soma area among the Sham, P7-8W, P7-16W, and Adult-8W groups, indicating that motor neuron survival and soma size in the lumbar spinal cord were largely preserved after injury (Figure 9A–C).

In contrast, Tuj1^+^ neurite architecture surrounding motor neurons was markedly altered after SCI. Neurite density was significantly reduced in both the neonatal and adult groups compared with the sham group, with a further decline in the P7-16W group relative to P7-8W mice (Figure 9D). Compared with the Sham, average branch length was substantially shortened in the P7-8W, P7-16W, and Adult-8W groups after injury, with the most severe reduction observed in the Adult-8W group (Figure 9E). Junction analysis further revealed dynamic remodeling of the local neuronal network: the number of neurite junctions was increased in the P7-8W group, but decreased in the P7-16W group, while remaining elevated in the Adult-8W group (Figure 9F). Together, these findings indicate that neonatal mice following SCI do not markedly affect lumbar motor neuron number, but may imply some reorganization of the surrounding neurite structure.

### 3.5. Partial Preservation of Presynaptic Markers Around Lumbar Motor Neurons in Neonatal Mice After SCI

**Figure 10:**
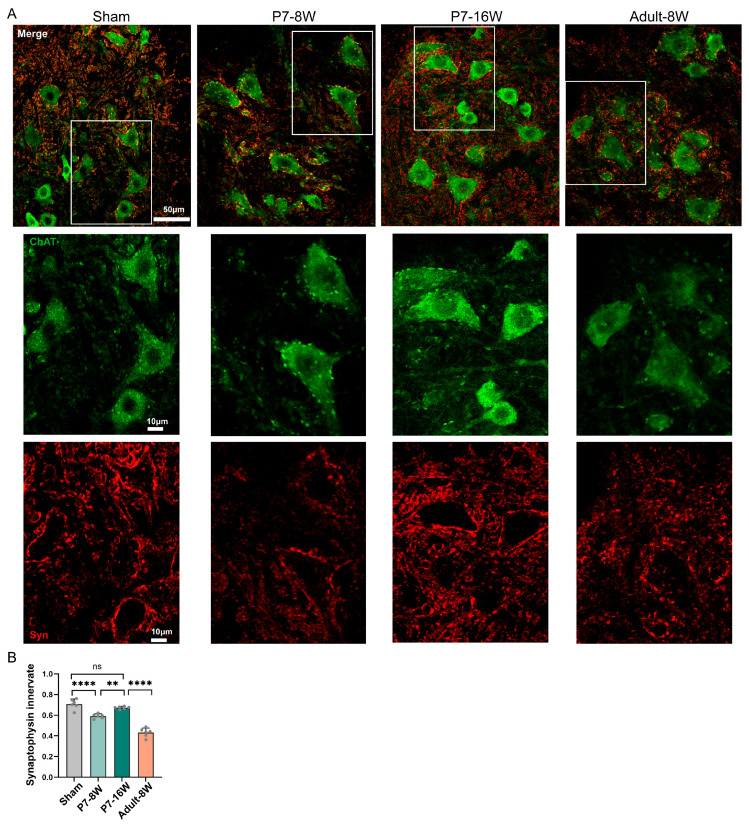
Synaptic coverage around lumbar motor neurons is partially restored in neonatal mice after complete spinal cord transection. (**A**) Representative immunofluorescence images showing ChAT-positive motor neurons (Green) and Syn-positive synaptic puncta (Red) in the lumbar ventral horn from Sham, P7-8W, P7-16W, and Adult-8W mice. ChAT-positive motor neurons are shown in green, and Syn-positive synaptic structures are shown in red. The boxed regions in the merged images indicate the areas shown at higher magnification in the corresponding single-channel images below. Scale bars: 50 μm in merged images; 10 μm in enlarged images. (**B**) Quantification of the Syn-positive rate around ChAT-positive motor neurons. Compared with Sham mice, the Syn-positive rate was reduced after neonatal SCI at P7-8W but partially recovered at P7-16W. Adult-8W mice showed a more pronounced reduction in Syn-positive rate compared with the P7-8W and P7-16W groups. Data are presented as mean ± SD. Syn innervation was compared among groups using one-way ANOVA followed by Tukey’s multiple-comparisons test, and multiple testing across related synaptic histological endpoints was controlled using the Holm–Šídák correction. n = 6, ** *p* < 0.01, **** *p* < 0.0001, ns indicates no significant difference.

To further assess presynaptic structure remodeling around lumbar motor neurons, we examined synaptophysin labeling in ChAT^+^ neurons of the lumbar spinal cord. Representative images showed a reduction in Syn^+^ puncta surrounding motor neuron soma after SCI, with a more pronounced loss in adult mice with SCI (Figure 10A). Quantitative analysis demonstrated that the proportion of presynaptic structure was significantly decreased in the P7-8W group compared with the sham group, but remained higher than that in the Adult-8W group. Notably, synaptophysin coverage in the P7-16W group was slightly increased relative to P7-8W and was not significantly different from sham levels, suggesting partial remodeling of the presynaptic structure around lumbar motor neurons over time (Figure 10B). Together, these data indicate that neonatal SCI is associated with transient synaptic loss around lumbar motor neurons, followed by partial re-establishment of presynaptic structure, in contrast to the more severe and persistent denervation observed in adult mice after injury.

### 3.6. Myelin-Associated Damage Was Relatively Mild in Neonatal Mice Following SCI

**Figure 11:**
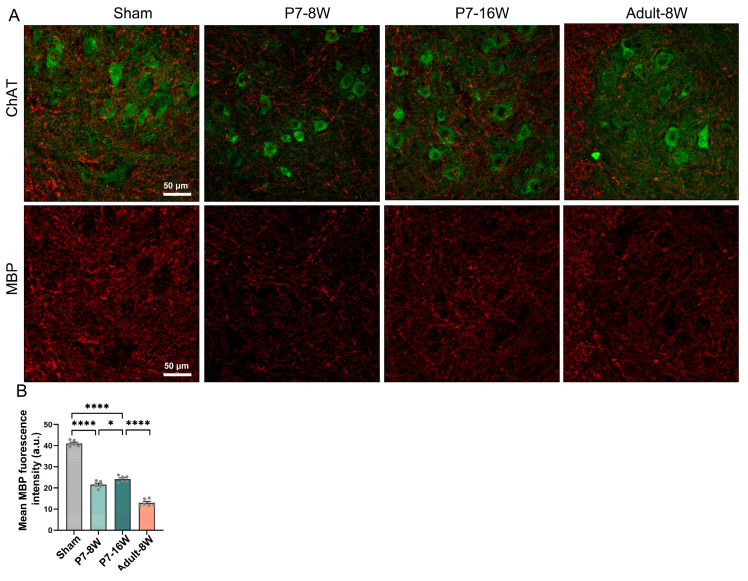
Myelin-associated signal in the lumbar ventral horn is relatively preserved in neonatal mice after complete spinal cord transection. (**A**) Representative immunofluorescence images showing ChAT-positive motor neurons (Green) and MBP-positive myelin-associated structures (Red) in the lumbar ventral horn from Sham, P7-8W, P7-16W, and Adult-8W mice. ChAT-positive motor neurons are shown in green, and MBP-positive myelin signal is shown in red. Scale bars: 50 μm. (**B**) Quantification of MBP mean fluorescence intensity in the lumbar ventral horn. MBP fluorescence intensity was reduced after spinal cord transection in both neonatal and adult mice compared with the sham mice. However, P7-16W mice showed significantly higher MBP intensity than P7-8W mice, and both neonatal SCI groups showed higher MBP intensity than Adult-8W mice, indicating relatively better preservation or recovery of myelin-associated structures in neonatal mice after injury. Data are presented as mean ± SD. Mean MBP fluorescence intensity was compared among groups using one-way ANOVA followed by Tukey’s multiple-comparisons test, and multiple testing across related histological/myelin-related endpoints was controlled using the Holm–Šídák correction. n = 6, * *p* < 0.05, **** *p* < 0.0001.

To examine whether the lumbar spinal cord remodeling in neonatal mice after SCI was accompanied by changes in myelin-associated structures, we assessed MBP immunoreactivity in the lumbar cord surrounding ChAT^+^ motor neurons. Representative images showed an overall reduction in MBP signal after SCI in both neonatal and adult mice following SCI compared with the sham group (Figure 11A). Quantitative analysis confirmed that mean MBP fluorescence intensity was significantly decreased in the P7-8W group and remained below sham levels in the P7-16W group, although a modest increase was observed over time. Importantly, MBP intensity in P7-8W and P7-16W groups was higher than that in the adult-8W group (Figure 11B). Combined with the EMG results, these findings suggest that the partial spontaneous recovery of motor function in neonatal mice is also related to the myelination process of the lumbar neural networks after SCI.

### 3.7. Excitatory and Inhibitory Synaptic Signals Around Lumbar Motor Neurons Undergo Dynamic Changes in Neonatal Mice with SCI

**Figure 12:**
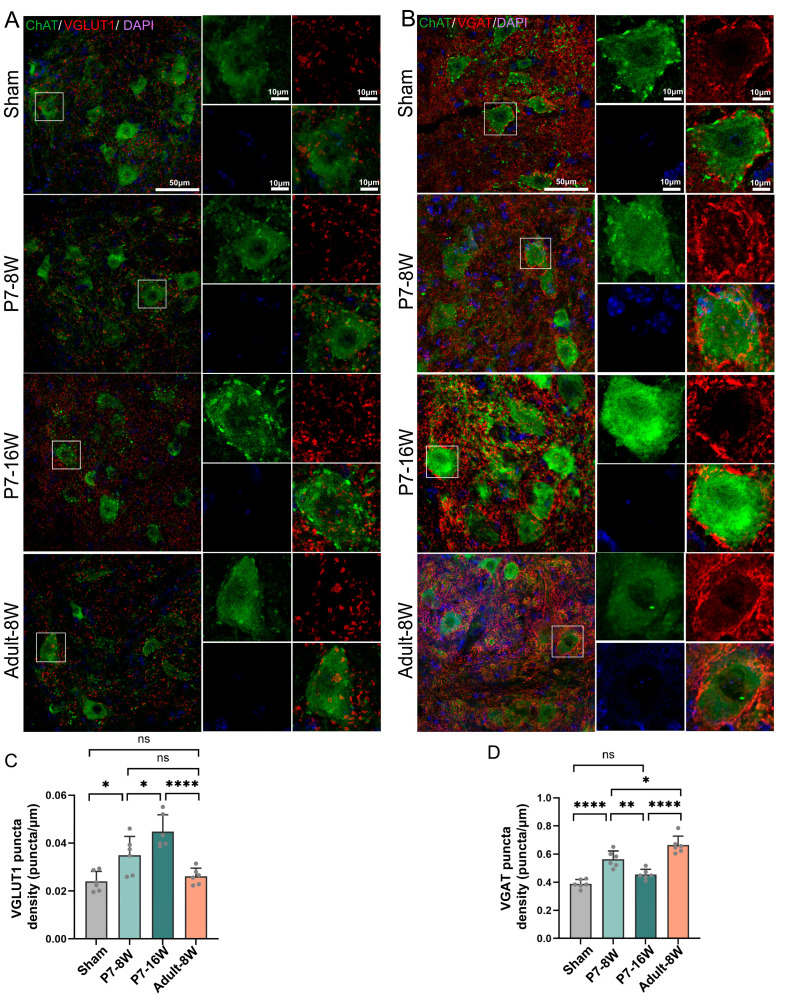
Changes in the excitatory–inhibitory synaptic signals of lumbar motor neurons. ((**A**,**B**) Representative images of ChAT (Green) co-labeled with VGLUT1 ((**A**), Red) and VGAT ((**B**), Red) immunofluorescence in the lumbar spinal cord of Sham, P7-8W, P7-16W, and Adult-8W groups. (**C**) Quantitative analysis of VGLUT1-positive synaptic density (puncta/μm) on the soma of ChAT-positive motor neurons. (**D**) Coverage of VGAT-positive synapses on ChAT-positive motor neurons. Data are presented as mean ± SD. VGLUT1 and VGAT puncta densities were analyzed using one-way ANOVA followed by the Tukey’s multiple-comparisons test, with the Holm–Šídák correction applied across related synaptic puncta-density endpoints. n = 6, * *p* < 0.05, ** *p* < 0.01, **** *p* < 0.0001, ns indicates no significant difference.

To further define the remodeling of synaptic properties around lumbar motor neurons after neonatal complete transection, we quantified excitatory and inhibitory signals using VGLUT1 and VGAT immunostaining, respectively. Representative images showed progressive changes in the distribution of both glutamatergic and GABA/glycinergic puncta around ChAT^+^ motor neurons across post-injury time points (Figure 12A,B). Quantitative analysis revealed that VGLUT1 puncta density was significantly increased in the P7-8W group compared with the sham group and was further elevated in the P7-16W group, indicating a progressive enhancement of excitatory signals surrounding lumbar motor neurons in neonatal mice after injury. In contrast, the Adult-8W group showed no significant increase relative to the sham group and exhibited lower VGLUT1 density than the P7-16W group (Figure 12C). Analysis of inhibitory signals showed that VGAT coverage was also significantly increased after injury. Compared with the sham, VGAT coverage was elevated in both P7-8W and P7-16W mice; however, the level in the P7-16W group was lower than that in the P7-8W group. Adult mice with SCI displayed the highest VGAT coverage among all groups (Figure 12D).

Together, these findings indicate that neonatal mice following SCI induce some remodeling of both excitatory and inhibitory synaptic signals around lumbar motor neurons. Importantly, compared with the adult mice after SCI, the neonatal mice with SCI were associated with a different pattern of synaptic reorganization, characterized by progressive restoration of excitatory signals together with moderate inhibitory coverage, which was accompanied by the partial recovery of hindlimb locomotion.

## 4. Discussion

The present study demonstrated that neonatal mice exhibit spontaneous partial recovery of hindlimb motor function following complete thoracic spinal cord transection. Compared with adult SCI mice subjected to the same injury, P7-injured mice showed partial improvement in BMS scores, gait parameters, and fine motor performance. Although locomotor recovery was not fully achieved, these findings suggest that the developmental stage plays a significant role in the capacity for spontaneous functional improvement following SCI. This observation aligns with previous studies that have reported greater plasticity in neonatal spinal cord injury models compared to those in adults [21,22].

Partial spontaneous recovery of hindlimb motor function was observed in neonatal mice following complete thoracic spinal cord transection, despite the absence of detectable supraspinal reconnection across the lesion site. Histological analysis showed a clear separation between the rostral and caudal spinal cord stumps, with no continuous NF200-positive axonal bridge detected across the lesion gap. Therefore, the recovered hindlimb output is more likely to be associated with these, including lumbar spinal circuits, peripheral neuromuscular components, and sensory-motor feedback loops, rather than direct descending supraspinal input [11,23,24,25]. Previous studies have shown that spinal locomotor circuits are capable of producing rhythmic motor patterns in isolated or deafferented conditions, supporting the concept that basic locomotor programs are embedded within the spinal cord itself [26,27]. The present findings are consistent with these reports.

Recovery was accompanied by structural changes within the distal lumbar spinal cord. Syn^+^ coverage was relatively preserved in neonatal mice compared with adult mice following SCI, while VGLUT1- and VGAT-positive puncta showed dynamic changes during the recovery period. In the spinal cord, VGLUT1 is associated with glutamatergic sensory afferent and propriospinal inputs relevant to motor control, and changes in VGLUT1-positive boutons on motoneurons have been widely used as a readout of synaptic reorganization after injury [28,29]. In adult mice with SCI, loss or maladaptive remodeling of glutamatergic synapses onto motoneurons is often linked to impaired motor output [11]. Meanwhile, VGAT-positive puncta remained above the sham level in neonatal mice with SCI, especially at the earlier time point. This may reflect a role for inhibitory signals in shaping rhythmic output, stabilizing motor patterns, or limiting inappropriate co-contraction during locomotor recovery. And adaptive neurotransmitter switching in SCI is helpful for restoring motor function independently of descending pathways [11]. However, because VGLUT1 or VGAT immunostaining does not determine the synaptic strength, timing, or cell-type origin, we cannot conclude that recovery results from a specific form of excitatory and inhibitory rebalancing. Collectively, the findings indicate that adult mice with SCI demonstrated a relatively low density of VGLUT1-positive puncta alongside a high density of VGAT-positive puncta, along with poor motor performance, suggesting a potential association between excitatory and inhibitory remodeling and functional outcomes. Nevertheless, it is important to note that this relationship remains correlative.

In addition to synaptic changes, neonatal-injured mice showed relatively preserved MBP immunoreactivity compared with adult-injured mice, suggesting a less severe disruption of myelin-associated structures in the distal spinal cord. This difference may reflect a developmental stage-dependent response of white matter to spinal cord injury, in which the neonatal spinal cord maintains a more permissive structural environment that is less susceptible to severe myelin disruption. Within this context, the MBP signal should be interpreted as an indicator of structural myelin integrity rather than a direct measure of axonal conduction or functional connectivity. Therefore, differences in the MBP immunoreactivity are more likely to reflect variation in tissue-level structural preservation rather than direct evidence of functional recovery mechanisms. Consistent with previous studies, myelin-associated molecules are known to regulate axonal stability and limit regenerative plasticity after CNS injury [30,31], and alterations in myelin structure have been associated with changes in conduction efficiency and motor performance [32,33]. In line with this, EMG recordings and neuromuscular junction analyses in the present study suggested partial preservation of peripheral motor output pathways after SCI; however, these measures may also be influenced by peripheral muscle and nerve condition. Together, these findings support an association between reduced myelin disruption and improved functional outcomes, while the causal contribution of myelin preservation to recovery remains to be established.

This study also has several limitations. First, the synaptic markers used here define structural apposition rather than functional synaptic strength; patch-clamp recording, optogenetic interrogation, or trans-synaptic tracing will be required to determine which premotor populations actually drive recovered stepping. Second, VGLUT1 and VGAT capture broad excitatory and inhibitory compartments but do not resolve the cellular identity of the reorganized interneuron. Third, our data establish an association rather than causation: they show that lumbar circuit remodeling accompanies spontaneous recovery, but not which molecular programs or cell types are necessary for it. Finally, the relatively small sample size and lack of a formal priori power calculation mean that the findings should be interpreted as descriptive and exploratory.

In summary, neonatal complete spinal cord transection is associated with partial spontaneous recovery of hindlimb motor function and accompanied by structural and electrophysiological changes in the distal lumbar motor system. These findings support a model in which the developing spinal cord retains a greater capacity for recovery-associated plasticity. Future studies using functional circuit interrogation, cell-type-specific manipulation, and electrophysiological approaches will be required to determine the causal contribution of these changes to locomotor recovery.

## 5. Conclusions

In conclusion, neonatal complete spinal cord transection was followed by partial spontaneous recovery of hindlimb motor function and was accompanied by structural and electrophysiological changes in the distal lumbar motor system. These findings support an association between neonatal injury, functional improvement, and lumbar remodeling, while highlighting the greater plasticity of the developing spinal cord.

## Data Availability

The original data presented in this study are openly available in the article. Further inquiries can be directed to the corresponding author.

## Abbreviations

The following abbreviations are used in this manuscript:

SCI	Spinal Cord Injury
ChAT	Choline Acetyltransferase
VGLUT1	Vesicular Glutamate Transporter 1
VGLUT2	Vesicular Glutamate Transporter 2
VGAT	Vesicular GABA Transporter
MBP	Myelin Basic Protein
EMG	Electromyography
AChR	Acetylcholine Receptor
NMJ	Neuromuscular Junction
Syn	Synaptophysin
